# 西达本胺联合PD-1单抗治疗Sézary综合征获得持续缓解1例

**DOI:** 10.3760/cma.j.issn.0253-2727.2022.02.016

**Published:** 2022-02

**Authors:** 冲 魏, 炎 张, 薇 张, 道斌 周

**Affiliations:** 中国医学科学院、北京协和医学院北京协和医院血液内科，北京 100730 Department of Hematology, Peking Union Medical College Hospital, Chinese Academy of Medical Sciences & Peking Union Medical College, Beijing 100730, China

患者，男，71岁，因“反复皮疹10年，加重伴淋巴结肿大1年余”就诊于北京协和医院血液内科。患者2010年出现手背、双肘、足踝部皮肤红斑，伴脱屑、瘙痒，外用糖皮质激素治疗有效，但仍反复出现皮疹。2018年患者出现全身弥漫性红斑，累及90％体表面积，瘙痒明显，并可触及腹股沟淋巴结肿大。患者2018年6月就诊于我院皮肤科，血常规大致正常；外周血细胞形态学：可见2％～4％异形单个核细胞，具有脑回状核，形态符合Sézary细胞；皮肤活检病理：表皮角化过度伴角化不全，少量淋巴细胞移入表皮，真皮浅层血管扩张，血管周围单一核细胞浸润；免疫组化：CD4（++），CD5（++）、CD7（+）、CD8（+）、CD30（个别+）、PD-1（+）；TCRβ重排阳性，诊断为蕈样肉芽肿（红皮病型，T4NxM0B0，ⅢA期）。先后予光疗（UVA、UVB）联合干扰素、糖皮质激素口服联合阿维A胶囊、低剂量甲氨蝶呤治疗，症状均无改善，并出现手足皲裂、瘙痒难耐。2019年6月就诊于我院血液科，血常规：WBC 13.33×10^9^/L，HGB 111 g/L，PLT 161×10^9^/L；乳酸脱氢酶 323 U/L。血细胞形态学：Sézary细胞占28.5％（计算Sézary细胞绝对值为3.86×10^9^/L）；外周血淋巴细胞亚群：CD4^+^/CD8^+^T细胞比值26.5；骨髓免疫分型：CD4/CD8比值16.8，主要表达CD2、CD3，不表达CD16、CD56、CD57。腹股沟淋巴结活检病理：符合皮病性淋巴结炎。PET-CT：双侧腋窝、腹股沟代谢增高淋巴结，直径1～3 cm，SUVmax 1.6～2.2。修订版皮肤T细胞淋巴瘤严重度加权评分（mSWAT）为109.5分。诊断：Sézary综合征（T4N1M0B2，ⅣA期）。2020年6月予患者西达本胺联合CHOEP方案（环磷酰胺+表柔比星+长春地辛+依托泊苷+泼尼松）化疗，共行3个疗程。化疗后患者症状一过性好转，2个疗程化疗后再次加重。此后换用西达本胺联合GDP方案（吉西他滨+顺铂+地塞米松）化疗，共行3个疗程。3个疗程化疗后患者皮肤症状无改善，血细胞形态学：Sézary细胞占20％。2019年11月患者被纳入本中心开展的信迪利单抗联合西达本胺治疗复发/难治性皮肤T细胞淋巴瘤前瞻性单臂临床研究（ClinicalTrials.gov注册号：NCT04296786），具体用药方案为：信迪利单抗200 mg/次，第1日；西达本胺20 mg，每周2次，每3周为1个疗程。第1～2个疗程治疗期间患者出现一过性皮肤红斑、水肿、瘙痒加重。3个疗程治疗后患者皮肤红斑、脱屑及瘙痒开始逐步改善。10个疗程治疗后评估，mSWAT评分降至40分，外周血Sézary细胞降至8％，无新发淋巴结肿大及脏器受累，综合评估疗效为部分缓解。目前患者治疗周期延长为每2个月应用1次，随诊至2021年6月，患者仍为持续缓解状态。

讨论：进展期蕈样肉芽肿和Sézary综合征的治疗仍非常具有挑战性，目前尚无标准的一线治疗方案。该阶段的治疗往往需要多学科协作，联合皮肤靶向治疗、生物反应调节剂（干扰素、维A酸）及全身系统治疗，疾病晚期往往需加用细胞毒药物的化疗。虽然细胞毒药物化疗的反应率较高，但通常疗效难以持久，且会带来显著不良反应。近年来，多种组蛋白去乙酰化酶（HDAC）抑制剂已获批用于治疗复发/难治性皮肤T细胞淋巴瘤，如罗米地辛、伏立诺他，其单药治疗的有效率为30％～40％。PD-1单抗用于复发/难治性外周T细胞淋巴瘤的有效率同样为30％～40％。体外研究、动物实验及临床研究均发现HDAC抑制剂与PD-1单抗具有一定协同效应。该患者既往应用多种治疗均未获得疾病缓解，后应用HDAC抑制剂西达本胺联合PD-1单抗，疾病部分缓解，且疗效相对持久。

